# Evaluation of dental arch dimensions in 12 year-old Vietnamese children - A cross-sectional study of 4565 subjects

**DOI:** 10.1038/s41598-019-39710-4

**Published:** 2019-02-28

**Authors:** Truong Manh Dung, Vo Truong Nhu Ngoc, Nguyen Hung Hiep, Truong Dinh Khoi, Vu Van Xiem, Thien Chu-Dinh, Artur Cieslar-Pobuda, Eleana Stoufi, Pau Loke Show, Yang Tao, Nguyen Duy Bac, Nguyen Van Ba, Quynh-Anh Le, Van-Huy Pham, Dinh-Toi Chu

**Affiliations:** 1 School of Odonto Stomatology, Hanoi Medical University, Hanoi, Vietnam; 2 Hanoi General Hospital of Traditional Medicine, Hanoi, Vietnam; 3 Institute for Research and Development, Duy Tan University, 03 QuangTrung, Danang, Vietnam; 4 Centre for Molecular Medicine Norway (NCMM), Nordic EMBL Partnership, University of Oslo, Oslo, Norway; 5 Harvard School of Dental Medicine, Boston, MA 02115 USA; 6 Department of Oral Medicine, University of Rwanda, Republic of Rwanda, Kigali, 20093 Rwanda; 7 Department of Chemical and Environmental Engineering, Faculty of Engineering, University of Nottingham Malaysia Campus, Jalan Broga, 43500 Semenyih, Selangor Darul Ehsan, Malaysia; 8 College of Food Science and Technology, Nanjing Agricultural University, Nanjing, 210095 China; 9 Vietnam Military Medical University, Hanoi, Vietnam; 10 AI Lab, Faculty of Information Technology, Ton Duc Thang University, Ho Chi Minh City, Vietnam; 11 Faculty of Biology, Hanoi National University of Education, Hanoi, Vietnam

**Keywords:** Dental pulp, Dentine

## Abstract

This study aimed to define the width and length of the dental arch in 12-year-old Vietnamese children, and to elucidate differences between genders and among ethnic groups. A cross-sectional study was conducted in 4565 12 years-old children from the 4 major ethnic groups in Vietnam (Kinh, Muong, Thai, and Tay), with a healthy and full set of 28 permanent teeth that had never had any orthodontic treatment and with no reconstructive materials at the measured points. The mean variables in all subjects were 36.39 mm for upper inter-canine width; 46.88 mm for upper inter-first molar width; 59.43 mm for upper inter-second molar width; 10.41 mm for upper anterior length; 32.15 mm for upper posterior length 1; 45.52 mm for upper posterior length 2; 28.31 mm for lower inter-canine width; 41.63 mm for lower inter-first molar width; 54.57 mm for lower inter-second molar width (LM2W); 7.06 mm for lower anterior length (LAL); 26.87 mm for lower posterior length 1 (LP1L); and 41.29 mm for lower posterior length 2. Significant differences in these parameters between genders were found in all ethnic groups, except for LAL in the Kinh and Thai groups, and LP1L in the Tay group. Significant ethnic differences were also found in almost all parameters except LM2W in both males and females. Taken together, the representative sizes of dental arches of 12-year-old Vietnamese children have been defined. Our data indicate that there are some variations in dental arch dimensions among ethnic groups and between genders.

## Introduction

The population in Vietnam has reached 98 million in November 2018, and nearly 2 million inhabitants are children at the age of 12 years^1^. Among the 54 ethnic groups, Kinh, Tay, Thai and Muong account for 92% of the total population, with Kinh people being the overwhelming majority (87%). The Tay, Thai and Muong ethnic groups occupy only 5% of the population, living in the mountainous areas of Northern Vietnam^1^.

The form and size of the dental arch have long been an essential data source not only for dentistry but also for other fields such as biology, anthropometry, painting and sculpture^2–4^. The dental arch is a curve that is usually described and classified by quantitative mathematical equations or qualitative geometric forms. Its shape and size come from the natural balance of the jaw bone, the alveolar bone, and the muscles around them, and may also be affected by factors such as heredity, growth of the bones, eruption, rotation and inclination of the teeth, and environment^5–10^. Defining the shape of the dental arch is the most important part of diagnosing and planning treatment in orthodontics for optimal aesthetics, functioning, and long-term stability^11^. Over the past century, dental arch forms have been studied intensively in the hope of increasing understanding of the common shape and size of the teeth in each race. These studies may have applications in many fields beyond dentistry, such as medicine or biology^3,4,7–9,12,13^.

Much research has focussed on the morphology and characterization of the dental arch at different ages, such as the studies of Ross-Powell *et al*. on the American population^14^, Aluko *et al*. on the dental arch widths of Nigerian children^15^, Bishara *et al*. son changes of arch width in subjects aged from 6 weeks to 45 years^16^, Hassani *et al*. on Kenyan children aged 12^5^, and Burris and Harris on American blacks and whites^17^. Most of these studies are however longitudinal studies aimed at tracking the growth of children’s teeth at each stage^18^. Lundström showed that changes in size and form actually occur during the growth of permanent dentition (12–18 years)^12^.The 12-year-old is in puberty when the shape and size of the bow has the most dramatic and systematic growth, especially changes in the anterior segment of the arch consisting of the incisors and canines), when all the deciduous dentition is replaced by the permanent one s^8,9,12,13,19^. According to Ross-Powell and Harris, almost all changes in the dental arch occurs at the age of twelve^20^. Variation in the size and shape of the tooth arch are due to a variety of factors, such as supra-articular enlargement in the maxillary or changes in the alveolar crest^7,8,20,21^. The changes are dramatic in the mixed dentition period, and gradually decrease as the child completes the permanent dentition^8,13,18^. Early intervention to the teeth, especially at the age of twelve when the shape of the arch has a certain stability, brings more benefits than later treatment. If bite defect and instability in the jaw are treated early, this results in a shorter course of treatment and the patient soon develops the desired teeth. Thus, this is an optimal stage for orthodontic intervention^6–8^.

In Vietnam, local researchers have studied the shapes and sizes of dental arches for generations in order to obtain more complete reference data for dental practice. However, these studies have been conducted on a small scale and in limited regions, and their results cannot fully represent the common index of the entire Vietnamese population. There have been no studies on dental arch index at age 12 in Vietnam, and therefore when conducting research regarding the dental arch index, Vietnamese scientists still have to apply the Caucasian dental arch model to their subjects. Although recently the Vietnamese have been switching over to the Chinese and Japanese models for more cultural accuracy, it is still necessary to conduct research to figure out the characteristic features and the standard values of the Vietnamese dental arch dimensions. In this study, we aim to define the representative sizes of the dental arch of 12-year-old Vietnamese children, to evaluate differences between genders and between the four major ethnic groups in Vietnam, and to compare the values with those for other countries at the same age.

## Materials and Methods

### Subjects

This cross-sectional study was conducted on 4565 12-year-old Vietnamese children from the four major ethnic groups (Kinh, Tay, Thai and Muong) out of a total of 6247 children in that age group. Children were screened to select eligible participants who were living in Vietnam, had Vietnamese grandparents, and were of good health with no birth defects or bad habits (e.g. intoxication or drug abuse), with 28 normal teeth, and without any diseases or currently undergoing any orthodontic treatment or facial surgery. Criteria for exclusion were a history of facial or orthodontic surgery, no healthy or full sets of teeth, and no full or permanent second molars. All samples were randomly selected from a national research project of the School of Odonto-Stomatology, Hanoi Medical University from the years 2016 to 2017.

### Data collection

From qualified participants, impressions of the maxillary and mandibular teeth were taken using alginate material s (Aroma, GC Corp, Tokyo, Japan) and poured with dental plaster stone (New Plastone II, GC Corp, Tokyo, Japan). After setting, the impression was inverted on a plastic mold containing plaster to get a base for the cast.

**Figure 1:**
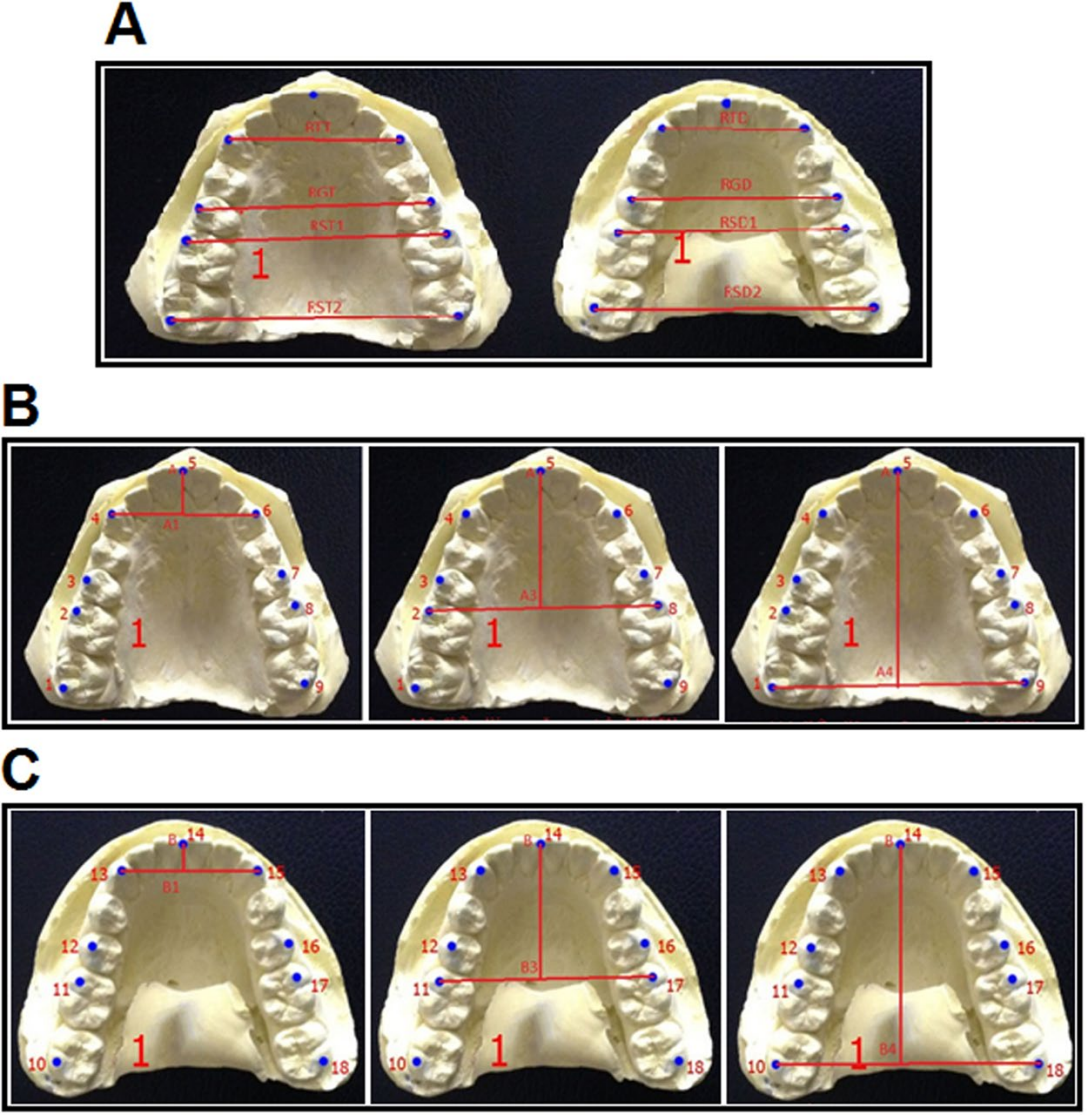
Studied dental arch dimensions. Index arch width (**A**), maxillary length (**B**) and mandibulaire length (**C**).

We measured the dental arch dimensions based on the studies of Engle *et al*.^22^, a method used previously by other authors^5,7,14,20^. All of the samples were measured by well-trained researchers using the same instrument (Mitutoyo CD-6) in the same natural light and room temperature conditions. Each sample was measured twice; if the difference between the two measurements was less than 0.2 mm the mean was taken as the final result and if it was more than 0.2 mm, the measurements were repeated. Training was made to achieve high consistency and Pearson coefficients were calculated between two measurements for each dimension to avoid errors. The selected landmarks were the points between the two incisors, the cusp tips of the permanent canine, and the tops of the permanent molars (Fig. 1 and Table 1). These are the common anatomical landmarks and are relatively easy to identify. From these landmarks, we measured the length and width of the jaw before making comparisons between sexes and ethnicities.

**Table 1:** Landmarks and measurements of dental arch dimensions.

Dimensions	Acronym	Definition
Upper inter-canine width	UCW	Distance between two cusp tips of left and right maxillary permanent canines of dental arch
Upper inter-first molar width	UM1W	Distance between two mesiolabial cusp tips of the right and left maxillary first permanent molars
Upper inter-second molar width	UM2W	Distance between two mesiolabial cusp tips of the right and left maxillary second permanent molars
Upper anterior length	UAL	Distance between centric point of upper incisal papilla to a horizontal line drawn between left and right cusp tips
Upper posterior length 1	UP1L	Distance between centric point of upper incisal papilla to a horizontal line drawn along the distal margins of the left and right first permanent molars
Upper posterior length 2	UP2L	Distance between centric point of upper incisal papilla to a horizontal line drawn along the distal margins of the left and right second permanent molars
Lower inter-canine width	LCW	Distance between two cusp tips of left and right mandibular permanent canines of dental arch
Lower inter- first molar width	LM1W	Distance between two mesiolabial cusp tips of the right and left mandibular first permanent molars
Lower inter- second molar width	LM2W	Distance between two mesiolabial cusp tips of the right and left mandibular second permanent molars
Lower anterior length	LAL	Distance between centric point of lower incisal papilla to a horizontal line drawn between left and right cusp tips
Lower posterior length 1	LP1L	Distance between centric point of lower incisal papilla to a horizontal line drawn along the distal margins of the left and right first permanent molars
Lower posterior length 2	LP2L	Distance between centric point of lower incisal papilla to a horizontal line drawn along the distal margins of the left and right second permanent molars

### Data analysis

Data were encoded and input using Epidata 3.0 software, and then were analyzed by the statistical software SPSS 23.0 (IBM, USA). Student’s t-tests, Mann-Whitney tests and Kruskal-Wallis tests were employed to compare the differences between/among groups classified by gender or ethnicity. A difference was considered statistically significant if P < 0.05.

## Results

In the total of 4565 study subjects, there were 2428 males (53–19%) and 2137 females (46.71%). We compared the dental arch dimensions in four ethnics (Table 2); the Kinh ethnic group was the majority (63.15%) (Table 1). The analysis showed that most indicators of males were statistically higher than those of females (P < 0.05), except for lower anterior length (LAL) in the Kinh and Thai ethnic groups and upper posterior length 1 (UP1L), upper anterior length (UAL), and Lower posterior length 1 (LP1L) in the Tay ethnic group (Table 3). We also compared the parameters according to ethnicity for males or females (Tables 4 and 5). In males, there were statistical differences (P < 0.001) among the 4 ethnic groups in all 12 indicators. For Muong males 7/12 indicators were smaller (UCW, UM1W, UM2W, UP2L, LM1W, loLM2W and LP2L) while for Tay males 5/12 were smaller (UAL, UP1L, LCW, LAL, and LP2L) (Table 4). In females, there were statistical differences among the 4 ethnic groups in 11/12 indicators (P < 0.05), except LM2W (Table 5); Tay females had 9/12 dimensions smaller (UCW, UM1W, UM2W, UAL, UP1L, UP2L, LCW, LAL, and LP1L) while Muong females had 2 smaller (LM1W and LP2L) (Table 5).

**Table 2:** The distribution of study subjects by genders and ethnicity.

Ethnic groups	Gender		n (%)
	Male n (%)	Female Total n (%) n	
Kinh	1589 (34.81)	1294 (28.35)	2883 (63.15)
Tay	167 (3.66)	168 (3.68)	335 (7.34)
Thai	351 (7.69)	314 (6.88)	665 (14.57)
Muong	321 (7.03)	361 (7.91)	682 (14.94)
Total	2428 (53.19)	2137 (46.81)	4565 (100.0)

**Table 3:** Comparison of dental arch dimensions between two genders in four ethnic groups.

Variable (mm)	Kinh			Tay			Thai			Muong			All ethnic groups		
	Male n = 1589 mean ± SEM	Female n = 1294 mean ± SEM	p	Male n = 167 mean ± SEM	Female n = 168 mean ± SEM	p	Male n = 351 mean ± SEM	Female n = 314 mean ± SEM	p	Male n = 321 mean ± SEM	Female n = 361 mean ± SEM	p	Male n = 2428 mean ± SEM	Female n = 2137 mean ± SEM	p
UCW	36.45 ± 2.36	35.68 ± 2.01	***	36.3 ± 1.92	35.06 ± 1.67	***	36.24 ± 2.45	35.8 ± 2.61	*	36.17 ± 2.32	35.42 ± 3.71	***	36.37 ± 2.34	35.61 ± 2.45	***
UM1W	54.78 ± 2.79	53.55 ± 2.67	***	54.65 ± 2.58	53.02 ± 2.79	***	55.16 ± 3.26	54.31 ± 3.21	***	54.6 ± 2.53	53.08 ± 2.5	***	54.8 ± 2.82	53.54 ± 2.76	***
UM2W	59.9 ± 3.09	59.53 ± 3.16	***	59 ± 3.54	57.52 ± 3.39	***	59.05 ± 3.93	57.92 ± 4.03	***	58.94 ± 3.14	57.86 ± 2.83	***	59.59 ± 3.29	58.85 ± 3.38	***
UAL	10.15 ± 2.45	9.36 ± 2.46	***	7.68 ± 2.69	7.42 ± 2.73	NS	9.86 ± 2.09	9.27 ± 1.99	***	8.53 ± 1.62	7.83 ± 1.31	***	9.72 ± 2.45	8.94 ± 2.37	***
UP1L	29.85 ± 4.03	29.01 ± 3.95	***	27.53 ± 3.34	27.28 ± 4.19	NS	28.96 ± 3.34	28.06 ± 3.43	***	28.46 ± 2.01	27.85 ± 2.25	**	29.38 ± 3.75	28.54 ± 3.71	***
UP2L	46.18 ± 3.14	44.19 ± 2.9	***	44.52 ± 4.75	41.98 ± 4.52	***	45.78 ± 3.3	43.07 ± 3.2	***	43.32 ± 1.59	42.85 ± 2.25	**	45.63 ± 3.3	43.62 ± 3.1	***
LCW	28.7 ± 2.43	27.58 ± 2.43	***	27.52 ± 3.43	26.83 ± 3.13	***	27.94 ± 2.69	26.91 ± 2.61	***	27.65 ± 2.4	26.93 ± 2.33	***	28.37 ± 2.59	27.31 ± 2.53	***
LM1W	46.75 ± 2.94	45.21 ± 3.03	***	47.16 ± 3.09	44.97 ± 2.94	***	47.92 ± 3.63	47 ± 3.52	***	47.11 ± 1.98	45.7 ± 2.32	***	46.99 ± 2.98	45.54 ± 3.06	***
LM2W	54.7 ± 3.36	52.6 ± 3.7	***	54.11 ± 3.38	52.7 ± 3.69	***	54.22 ± 3.88	53.13 ± 3.73	***	54.07 ± 2.93	52.61 ± 3.07	***	54.51 ± 3.4	52.69 ± 3.61	***
LAL	6.8 ± 2.3	6.69 ± 2.23	NS	5.1 ± 2.34	4.31 ± 2.29	***	6.79 ± 2.42	6.71 ± 2.71	NS	5.44 ± 1.37	5.16 ± 1.2	*	6.5 ± 2.3	6.25 ± 2.32	***
LP1L	25.89 ± 4.2	25.11 ± 4.01	***	23.29 ± 4.59	22.63 ± 3.23	NS	26.13 ± 2.84	24.66 ± 3	***	24.28 ± 1.54	23.4 ± 2.29	***	25.53 ± 3.89	24.56 ± 3.67	***
LP2L	41.94 ± 3.87	41.17 ± 3.54	***	40.96 ± 6.04	39.26 ± 4.15	***	40.09 ± 3.54	38.96 ± 4.12	***	39.63 ± 2.17	38.7 ± 2.48	***	41.3 ± 3.95	40.27 ± 3.7	***

P was calculated using Mann-Whitney test; NS: Not significant; *P < 0.05, **p < 0.01, ***P < 0.001.

**Table 4:** Difference of dental arch dimensions according to four ethnic groups in male subjects.

Variable (mm)	Kinh ethnic n = 1589 mean ± SEM	Tay ethnic n = 167 mean ± SEM	Thai ethnic n = 351 mean ± SEM	Muong ethnic n = 321 mean ± SEM	p
UCW	36.45 ± 2.36	36.3 ± 1.92	36.24 ± 2.45	36.17 ± 2.32	*
UM1W	54.78 ± 2.79	54.65 ± 2.58	55.16 ± 3.26	54.6 ± 2.53	*
UM2W	59.9 ± 3.09	59 ± 3.54	59.05 ± 3.93	58.94 ± 3.14	***
UAL	10.15 ± 2.45	7.68 ± 2.69	9.86 ± 2.09	8.53 ± 1.62	***
UP1L	29.85 ± 4.03	27.53 ± 3.34	28.96 ± 3.34	28.46 ± 2.01	***
UP2L	46.18 ± 3.14	44.52 ± 4.75	45.78 ± 3.3	43.32 ± 1.59	***
LCW	28.7 ± 2.43	27.52 ± 3.43	27.94 ± 2.69	27.65 ± 2.4	***
LM1W	46.75 ± 2.94	47.16 ± 3.09	47.92 ± 3.63	47.11 ± 1.98	***
LM2W	54.7 ± 3.36	54.11 ± 3.38	54.22 ± 3.88	54.07 ± 2.93	***
LAL	6.8 ± 2.3	5.1 ± 2.34	6.79 ± 2.42	5.44 ± 1.37	***
LP1L	25.89 ± 4.2	23.29 ± 4.59	26.13 ± 2.84	24.28 ± 1.54	***
LP2L	41.94 ± 3.87	40.96 ± 6.04	40.09 ± 3.54	39.63 ± 2.17	***

P was calculated using Kruskal-Wallis test; NS: Not significant; *P < 0.05, **p < 0.01, ***P < 0.001.

**Table 5:** Difference of dental arch dimensions according to four ethnic groups in female subjects.

Variable (mm)	Kinh ethnic n = 1294 mean ± SEM	Tay ethnic n = 168 mean ± SEM	Thai ethnic n = 314 mean ± SEM	Muong ethnic n = 361 mean ± SEM	p
UCW	35.68 ± 2.01	35.06 ± 1.67	35.8 ± 2.61	35.42 ± 3.71	***
UM1W	53.55 ± 2.67	53.02 ± 2.79	54.31 ± 3.21	53.08 ± 2.5	***
UM2W	59.53 ± 3.16	57.52 ± 3.39	57.92 ± 4.03	57.86 ± 2.83	***
UAL	9.36 ± 2.46	7.42 ± 2.73	9.27 ± 1.99	7.83 ± 1.31	***
UP1L	29.01 ± 3.95	27.28 ± 4.19	28.06 ± 3.43	27.85 ± 2.25	***
UP2L	44.19 ± 2.9	41.98 ± 4.52	43.07 ± 3.2	42.85 ± 2.25	***
LCW	27.58 ± 2.43	26.83 ± 3.13	26.91 ± 2.61	26.93 ± 2.33	***
LM1W	45.21 ± 3.03	44.97 ± 2.94	47 ± 3.52	45.7 ± 2.32	***
LM2W	52.6 ± 3.7	52.7 ± 3.69	53.13 ± 3.73	52.61 ± 3.07	NS
LAL	6.69 ± 2.23	4.31 ± 2.29	6.71 ± 2.71	5.16 ± 1.2	***
LP1L	25.11 ± 4.01	22.63 ± 3.23	24.66 ± 3	23.4 ± 2.29	***
LP2L	41.17 ± 3.54	39.26 ± 4.15	38.96 ± 4.12	38.7 ± 2.48	***

P was calculated using Kruskal-Wallis test; NS: Not significant; *P < 0.05, **p < 0.01, ***P < 0.001.

## Discussion

Vietnam’s population has reached nearly 98 million people, of which around 2 million are 12-year-old children^1^. The inhabitants of Viet Nam are ethnically quite diverse and the population in each ethnic group, especially the Kinh majority, has increased dramatically. The age group on which we conducted this study was that at which the teeth are relatively full and stable on the bones, which facilitated the study of their morphological characteristics. Since this was a study of dental arch dimensions, the criteria for the choice of the patients had to be strict to avoid deviations. For example, there were facial requirements such as no facial deformities, no surgery or orthodontics, no history of traumatic injury or congenital malformations. In addition, the criteria for teeth and dental arch were very strict as the participants had to have a full set of 28 permanent teeth, and no restoration or major damage that changed the dimensions of the teeth which can occur in children 12 years of age. Because it is difficult to measure the dimensions of teeth and dental arch accurately when the teeth are in the mouth, we used the method of measurements on casts which are widely applied^23,24^.

Our research aimed to determine the average dental arch dimensions of Vietnamese children at the age of 12 and to detect any differences in these indicators between the two genders and among local ethnic majorities, and later to compare them with other ethnic groups in the world. We observed that most indicators of males were statistically higher than those of females in our study population. This is consistent with the study done by Ross-Powell *et al*.^20^ on black and white children aged 3 to 18 years; these authors found that the dimensions of the lower teeth were similar between male and female children aged 3 to 10, but at the onset of puberty the difference in arch sizes between genders was statistically significant, higher in males than females. Bishara *et al*.^16^ in their study on subjects aged 6 weeks to 45 years found that the length of the male arch was significantly greater than that of the female, possibly because the arch dimensions begin to change in children at 10–12 years and the boys begin to grow faster than girls. The gender difference between these indexes also corresponds to the different body development between men and women. In men, most anthropometric measures such as average height, average weight, or index of head area are larger than women. Thus, to ensure the harmonious development and balance of the head and face as well as the entire body, the males’ dental arch dimensions are usually larger than those of females, which is consistent with human evolution^20^.

The average indicators of Vietnamese children aged 12 were also similar to those found in studies of Chinese and Kenyan children at the age of 12^5,25^. Compared to the study done by Hassanali *et al*.^5^ on 12-years-old Kenyans, only upper inter-canine width is similar in our study while the remaining indicators are different; especially, the upper and lower jaw of the children in Kenya were significantly longer than the children in Vietnam (Table 6). Compared to upper and lower intercanine width and anterior length of American blacks aged 12 in the study of Ross-Powell *et al*.^20^, we found that the width between the canines (front width) in the upper and lower jaw and the frontal length in the upper and lower jaw of the 12-year-old Vietnamese group were smaller than those of black American children at the same age. Therefore, the jaws of 12-year-old Vietnamese children are smaller and shorter than those of Caucasian (American) children. However, the length of the lower jaw in both males and females of the Vietnamese group is larger than that of Caucasian children (Table 7). These indicators were considerably larger than those for 12-year-old Brazilian children^26^. The results of Louly *et al*. for dental arch length and width were similar to those for our 12-year-old children, but their samples showed mixed dentition while ours chose 12-year-olds who had replaced all their teeth and had the second molars. Moreover, Louly *et al*.’s measurements on the first molar were not at the cusp tip but rather at the central groove of the teeth, and therefore they cannot be compared with our studies. Comparing to 12-year-old South Chinese of the same race (Mongoloid) and geographic proximity (border crossings), we found that only the width between the two first molars of 12-year-old Chinese was narrower than for Vietnamese while the rest of the parameters of tooth width are very similar, especially the canine width index and the width between the two second molars of the two jaws. It is clear that differences in racial origins as well as differences in evolutionary traits between different races, together with environmental factors, can explain the statistically significant differences in the dimensions of the dental arch in children from different continents with distinct genetics and diverse living conditions. Furthermore, when studying children 12 years of age one needs to consider that they are in a period of significant developmental change.

**Table 6:** Comparison of dental arch dimensions between different studies.

Variable (mm)	Our study (2017) n = 4565	Hassanali^5^ n = 173	Ling^25^ n = 358		
	mean ± SEM	mean ± SEM	P	mean ± SEM	P
UCW	36.01 ± 2.42	36.00 ± 2.4	**NS**	36.16 ± 0.94	NS
LCW	27.88 ± 2.61	26.30 ± 2.9	***	27.97 ± 0.57	**NS**
UM1W	54.21 ± 2.86	55.50 ± 2.50	***	53.75 ± 0.97	**
LM1W	46.31 ± 3.1	48.30 ± 2.50	***	45.64 ± 0.69	***
UM2W	59.24 ± 3.35			58.84 ± 1.09	*
LM2W	53.66 ± 3.61			53.52 ± 1.20	**NS**
UP1L	28.99 ± 3.75	36.7 ± 2.50	***		
LP1L	25.08 ± 3.82	31.5 ± 2.90	***		

P was calculated using student t test; NS: Not significant, *P < 0.05, **p < 0.01, ***P < 0.001.

**Table 7:** Comparison of dental arch dimensions with Ross-Powell’s study.

	Variable (mm)	Our study (2017)			Ross-Powell^20^			P
		n	Mean	SEM	n	Mean	SEM	
Female	UCW	2137	35.61	2.45	25	36.70	2.80	***
	LCW	2137	26.93	2.33	26	28.,60	1.9	***
	UAL	2137	8.94	2.37	25	11.10	1.90	***
	LAL	2137	6.25	2.32	25	4.80	1.30	***
Male	UCW	2428	36.37	2.34	23	36.90	2.30	**
	LCW	2428	27.65	2.4	25	28.40	2.10	***
	UAL	2428	9.72	2.45	23	11.10	1.7	***
	LAL	2428	6.5	2.3	25	5.,3	1.5	***

P was calculated using student t test; NS: Not significant, *P < 0.05, **p < 0.01, ***P < 0.001.

## Conclusions

Our study of 4565 Vietnamese children of four ethnic groups (Kinh, Tay, Thai and Muong) showed that most dental arch indicators in males were statistically significantly higher than those in females. Regarding the ethnicity, there were statistical differences in both males and females. In Muong males and females 7/12 and 2/12 indicators were smaller, respectively, compared to males and females in other ethnics, while in Tay males and females 5/12 and 9/12 dimensions were smaller, respectively, compared to these genders in other races. In comparison to other ethnic groups, 12-year-old Vietnamese children had similar dimensions of the upper and lower intercanine and intermolar width to children in the same age group in South China. However, the average upper posterior length 1 and lower posterior length 1 were shorter than those in Africans (Kenyan) and Caucasian (American blacks aged 12). The 12-year-old Vietnamese have a narrower and shorter dental arch than Caucasian children, especially the maxillary, and they need earlier orthodontic intervention.

### Ethical approval

All procedures performed in studies involving human participants were in accordance with the ethical standards of the institutional and/or national research committee and with the 1964 Helsinki declaration and its later amendments or comparable ethical standards. Our study was a part of the national project entitled “Vietnamese Characteristics of Craniofacial anthropometry to apply in medicine” (coded ĐTĐL.CN.27/16) which was approved by Hanoi Medical Council of Ethics for Biomedical Research in 2016 (IRB code - VN01001).

### Informed consent

Informed consents were obtained from the parents of all children included in the study.
