# Author Correction: Evaluation of dental arch dimensions in 12 year-old Vietnamese children - A cross-sectional study of 4565 subjects

**DOI:** 10.1038/s41598-019-53311-1

**Published:** 2019-11-06

**Authors:** Truong Manh Dung, Vo Truong Nhu Ngoc, Nguyen Hung Hiep, Truong Dinh Khoi, Vu Van Xiem, Thien Chu-Dinh, Artur Cieslar-Pobuda, Eleana Stoufi, Pau Loke Show, Yang Tao, Nguyen Duy Bac, Nguyen Van Ba, Quynh-Anh Le, Van-Huy Pham, Dinh-Toi Chu

**Affiliations:** 1 School of Odonto Stomatology, Hanoi Medical University, Hanoi, Vietnam; 2 Hanoi General Hospital of Traditional Medicine, Hanoi, Vietnam; 3 Institute for Research and Development, Duy Tan University, 03 QuangTrung, Danang, Vietnam; 4 Centre for Molecular Medicine Norway (NCMM), Nordic EMBL Partnership, University of Oslo, Oslo, Norway; 5 Harvard School of Dental Medicine, Boston, MA 02115 USA; 6 Department of Oral Medicine, University of Rwanda, Republic of Rwanda, Kigali, 20093 Rwanda; 7 Department of Chemical and Environmental Engineering, Faculty of Engineering, University of Nottingham Malaysia Campus, Jalan Broga, 43500 Semenyih, Selangor Darul Ehsan Malaysia; 8 College of Food Science and Technology, Nanjing Agricultural University, Nanjing, 210095 China; 9 Vietnam Military Medical University, Hanoi, Vietnam; 10 AI Lab, Faculty of Information Technology, Ton Duc Thang University, Ho Chi Minh City, Vietnam; 11 Faculty of Biology, Hanoi National University of Education, Hanoi, Vietnam

Correction to: *Scientific Reports* 10.1038/s41598-019-39710-4, published online 28 February 2019

The original version of this Article contained an error in Affiliation 10, which was incorrectly given as ‘AI Lab, Faculty of Information Technology, Ton Duc Thang University, Ho Chi Minh City, Vietnam, Ho Chi Minh City, Vietnam’. The correct affiliation is listed below:

AI Lab, Faculty of Information Technology, Ton Duc Thang University, Ho Chi Minh City, Vietnam

This error has now been corrected in the HTML and PDF versions of the Article.

